# A Comparative Study of the In Vitro Intestinal Permeability of Pinnatoxins and Portimine

**DOI:** 10.3390/md23010026

**Published:** 2025-01-07

**Authors:** Rachelle Lanceleur, Vincent Hort, Marion Peyrat, Denis Habauzit, Andrew I. Selwood, Valérie Fessard

**Affiliations:** 1 Toxicology of Contaminants Unit, Fougères Laboratory, ANSES (French Agency for Food, Environmental and Occupational Health & Safety), 35306 Fougères, France; rachelle.lanceleur@anses.fr (R.L.); denis.habauzit@anses.fr (D.H.); 2 Pesticides and Marine Biotoxins Unit, Laboratory for Food Safety, ANSES (French Agency for Food, Environmental and Occupational Health & Safety), 94701 Maisons-Alfort, France; vincent.hort@anses.fr (V.H.); marion.peyrat@anses.fr (M.P.); 3 Cawthron Institute, Private Bag 2, Nelson 7042, New Zealand; andy.selwood@cawthron.org.nz

**Keywords:** *Vulcanodium rugosum*, phycotoxins, Papp, TEER, barrier integrity, cyclic imines, QSAR predictions

## Abstract

The pinnatoxins (PnTXs) and portimines, produced by *Vulcanodinium rugosum*, have been detected in several countries, raising concerns for human health. Although no human poisoning from these toxins has been reported so far, they have been shown to distribute throughout the rodent body after oral administration. Therefore, we investigated the impact of PnTX analogs (PnTX-A, -E, -F, -G, and -H) and portimine (8, 16, and 32 ng/mL) on intestinal barrier integrity and their oral bioavailability using human Caco-2 cell monolayers treated for 2, 6, and 24 h. Our results demonstrated that all of the toxins could impair barrier integrity after 24 h, with differences observed for PnTX-A, -E, and -F, as well as portimine, the most potent of all. While PnTX-A and -E exhibited poor permeability, the other PnTXs were more penetrative, with a Papp > 1.5 × 10^−6^ cm·s^−1^. Portimine was the only toxin displaying both a time- and concentration-dependent passage, likely involving a passive diffusion process. The experimental results were compared to predictions obtained by QSAR tools. Although only qualitative, our results suggest that some of these compounds may be more likely to be distributed throughout the body. Further in vivo studies are required to estimate oral bioavailability and potential public health concerns.

## 1. Introduction

*Vulcanodium rugosum* is a dinoflagellate that was discovered in 2010 during biomonitoring surveys conducted at Ingril Lagoon, France [1]. This toxic microalgae has been shown to produce several toxins of the cyclic imines group, including pinnatoxins (PnTXs) and portimines. Like many other phycotoxins, these toxins may accumulate in shellfish and undergo biotransformation, leading to the formation of numerous analogs to which consumers may be exposed through dietary exposure.

**Figure 1:**
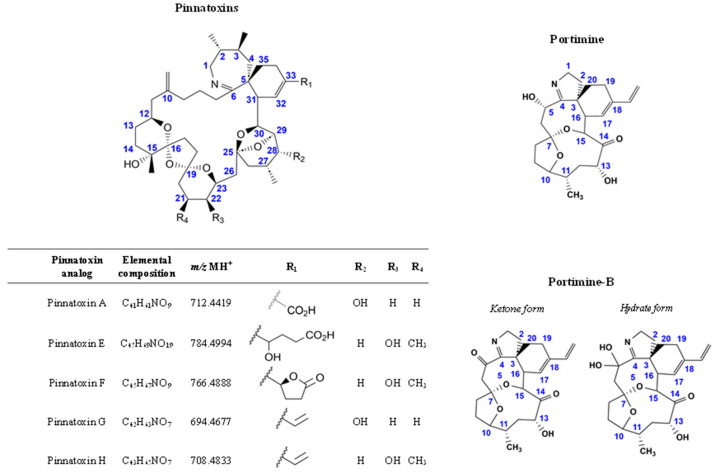
Structures of the toxins tested in this study and portimine-B.

Five pinnatoxin analogs are known to be directly produced by *V. rugosum*: PnTX-A, -E, -F, -G, and -H (Figure 1). Different toxin profiles were reported worldwide, dominated by PnTX-H in Qatar and China [2,3,4], PnTX-E and -F in New Zealand [5], PnTX-A, -E, -F, and -G in Australia [5], PnTX-E and PnTX-F in Cuba [6], and PnTX-G in France and Japan [7,8]. In shellfish, PnTXs have been detected in various species from New Zealand, Australia, Canada, Chile, Mozambique, and several European countries [9,10,11,12,13,14,15,16].

To date, the maximum PnTX level measured in shellfish was 1244 µg PnTX-G/kg in mussels from France in 2010 [17]. This raised concerns that such high concentrations may pose a threat to human health. In 2020, the French Agency for Food, Environmental, and Occupational Health and Safety (Anses) established a provisional acute health-based guidance value of 0.13 μg PnTX-G/kg body weight per day and proposed a maximum tolerable concentration of 23 μg PnTX-G/kg in shellfish [18]. Nevertheless, to date, the presence of PnTXs in shellfish has not been linked to human poisoning [19]. While the absence of effects may be reliable for acute toxicity, the potential relationship between repeated oral exposure to PnTX-contaminated shellfish and long-term effects would be likely more difficult to pinpoint.

Both in vitro and in vivo studies concluded that PnTX-F exhibits higher potency compared to PnTX-G and -E [20,21]. Indeed, oral toxicity studies showed the following potency ranking: PnTX-F > PnTX-G ~ PnTX-H ≫ PnTX-E. While PnTX-F and -G were also the most potent by the intraperitoneal route, PnTX-E toxicity was similar to PnTX-G, while PnTX-H and PnTX-A showed less potency [18]. Pinnatoxins have been classified as fast-acting toxins, generating neurotoxic effects and respiratory failure in mice, with death occurring only a few minutes after intraperitoneal injection [3,22,23]. These toxins act as antagonists of muscle-type nicotinic acetylcholine receptors (nAChRs) [20,24] by blocking synaptic transmission at neuromuscular junctions. Recently, using a radioactive compound, the crossing of the intestinal, blood–brain, and placental barriers by PnTX-G has been demonstrated in mice, with toxin distribution to the liver, kidney, and spleen [25].

*V. rugosum* also produces portimines, another group of cyclic imine toxins. In 2013, portimine was isolated from a *V. rugosum* isolate collected in New Zealand [26], and its structure was elucidated (Figure 1). More recently, another analog called portimine-B was discovered [27]. The original structure was misassigned and has recently been corrected [28,29]. Like many other marine biotoxins, such as PnTX-G, portimine may form a series of fatty acid esters conjugates (*n* = 13) [30], probably resulting from shellfish metabolism. Portimine can be produced in large quantities by *V. rugosum*. Nevertheless, this toxin seems to have a lower accumulation capacity in shellfish compared to PnTX-G [6,8]. Recent contamination data from France seem to confirm these results, as portimine concentrations in shellfish are lower than those of PnTX-G [31,32]. Unlike PnTXs, portimine was shown to be not very toxic by acute intraperitoneal exposure of mice [26]. In fact, portimine induces apoptosis, as observed by several methods [26,28,33]. Portimine-B also induces apoptosis in cancer cells but with less activity [27,28]. However, it did not affect low-proliferating cell systems, such as human peripheral blood mononuclear cells [28] and differentiated liver HepaRG cells [34]. Due to a lack of data, portimines were not included in the Anses appraisal, and the need for additional toxicological studies was highlighted [18].

Since these recently discovered toxins are distributed worldwide and can accumulate in shellfish, their potential impact on human health raises questions that must be explored in greater depth. First, it is important to investigate their fate after ingestion. Therefore, we considered an easy and quick strategy to study and compare the potency of several PnTX analogs and portimine for local effects on the intestine (intestinal integrity), as well as their ability to cross the intestinal barrier and reach systemic organs such as the liver. For this purpose, we assumed that an original approach to obtain qualitative information, with an affordable cost and without animal testing, is to use a simple Caco-2 cell monolayer and address these endpoints according to the European Centre for the Validation of Alternative Methods guidance document n°142 [35,36]. Although this cell line is derived from a human colorectal carcinoma, it can differentiate into enterocytic-like cells [37,38]. Moreover, it has been recognized as a valuable tool by the European Medicines Agency and the Food and Drug Administration for predicting the bioavailability of drugs [39,40]. Consequently, this system has been used to study the kinetics of numerous compounds [41,42,43]. In fact, it has shown an ability to accurately predict the intestinal permeability of certain drugs at an early stage [44].

## 2. Results

### 2.1. Trans-Epithelial Electrical Resistance (TEER)

**Figure 2:**
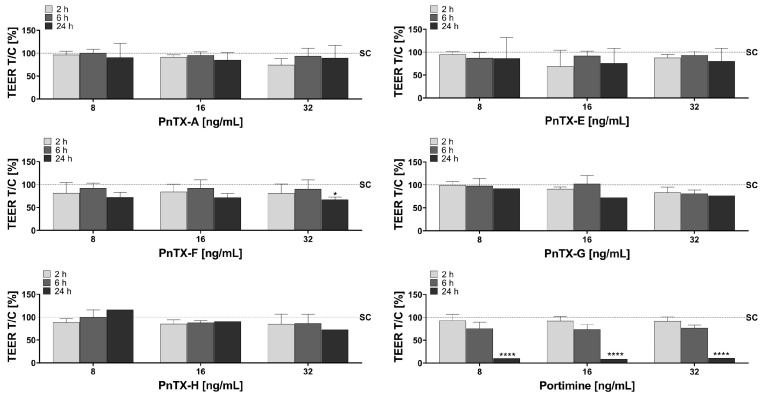
Trans-epithelial electrical resistance (TEER) of differentiated Caco-2 cells after incubation with PnTX-A, PnTX-E, PnTX-F, PnTX-G, PnTX-H, and portimine (8, 16, and 32 ng/mL) during 2 h (light grey), 6 h (grey), and 24 h (dark grey) or solvent control (SC, 1.5% MeOH, or 1.5% acetic acid/acetonitrile, dashed line). Results are depicted as mean ± standard deviation of 3 biological replicates (excluding PnTX-G, PnTX-H, and portimine at 24 h, for which only two replicates were available) and are normalized to the solvent control (dashed line). Statistical significance between the solvent control and the treated samples is indicated by * *p* ≤ 0.05 and **** *p* ≤ 0.0001.

TEER was used for measuring intestinal barrier integrity. All toxins at all concentrations (8, 16, and 32 ng/mL) had negligible impact on TEER values at 2 h and 6 h (Figure 2). However, some effects were observed at 24 h. For portimine, a significant decrease (10.02 ± 0.96% of control value) was obtained at 24 h for all of the concentrations. A slight non-significant decrease of TEER was observed for PnTX-F at 8 and 16 ng/mL, which became significant at 32 ng/mL (67 ± 5.6% of the control value). A slight, but non-significant, decrease in TEER was also observed at 16 and 32 ng/mL for PnTX-E and PnTX-G, as well as at 32 ng/mL for PnTX-H. PnTX-A was the only toxin showing no effect on TEER, irrespective of time and concentration.

### 2.2. Paracellular Permeability: Lucifer Yellow (LY)

**Figure 3:**
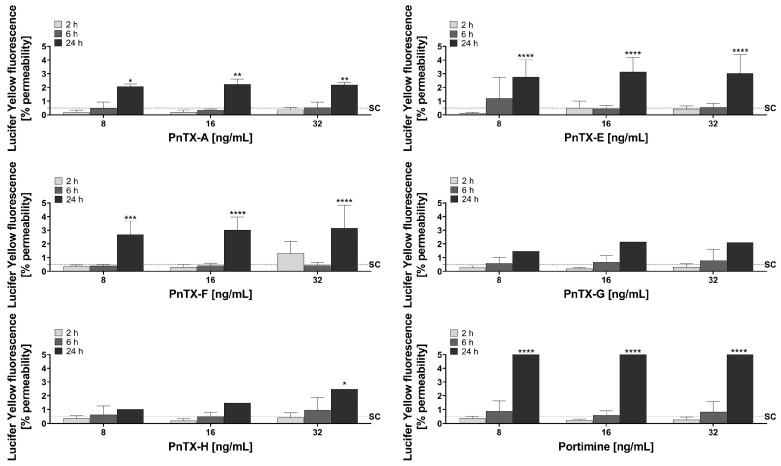
Lucifer Yellow fluorescence intensity in the basolateral compartment after incubation with PnTX-A, PnTX-E, PnTX-F, PnTX-G, PnTX-H, and portimine (8, 16, and 32 ng/mL) during 2 h (light grey), 6 h (grey), and 24 h (dark grey) compared to the solvent control (SC, 1.5% MeOH, or 1.5% acetic acid/acetonitrile, dashed line). Results are depicted as mean ± standard deviation of at least three biological replicates (excluding PnTX-G, PnTX-H, and portimine at 24 h, for which only two replicates were available). Statistical significance between the solvent control and the treated samples is indicated by * *p* ≤ 0.05, ** *p* ≤0.01, *** *p*≤ 0.001 and **** *p* ≤ 0.0001.

Barrier integrity was investigated by the paracellular passage of Lucifer Yellow. When the tight junctions are impaired, this compound can pass from the apical to the basolateral compartment. The measurement of LY paracellular permeability shows a negative correlation with TEER. Specifically, when TEER decreases due to the impairment of tight junctions, LY can cross the Caco-2 monolayer, leading to an increase in LY permeability. In the solvent control, only 0.518 ± 0.017% of the dye crossed the intestinal cell monolayer, indicating that the cell monolayer was intact (Figure 3). However, at 24 h, we observed that all toxins increased the passage of LY, some being more potent than others. A significant increase in LY permeability was observed for all concentrations with PnTX-A, PnTX-E, PnTX-F, and portimine. PnTX-G and PnTX-H had a lower effect, with a significant increase observed only at 32 ng/mL for PnTX-H.

Overall, the results of LY permeability and TEER measurements showed a good correlation, although LY permeability appeared to be slightly more sensitive. Considering the impact of all the toxins on LY permeability at 24 h, the samples collected at this time point were not dosed, as the crossing could be attributed to the impairment of the monolayer. 

### 2.3. Toxin Crossing 

Regardless of the concentration loaded, the amount of PnTX-A and PnTX-E crossing the intestinal barrier in vitro was very low (<4%) (Table 1). This was consistent with the minimal decrease in toxin levels detected over time in the apical compartment, regardless of the initial concentration (Table S2). However, the amount of PnTX-E in the apical compartment showed a slight decrease (approximately 15% for all the tested concentrations) at 2 h, which returned to approximately 100% at 6 h. This could suggest the involvement of an efflux transporter that pumps the toxin back to the lumen after it is taken up by the intestinal cells.

For PnTX-F, the percentage of toxin that crossed the barrier was around 12% at 2 h and increased approximately to 22% at 6 h regardless of the loaded concentration. However, the amount of PnTX-F in the apical compartment did not show any significant decrease (100 to 90% of the loaded amount) at either time point or any of the three concentrations. It should be noted that the T0 concentrations were lower than the expected loaded amount, with a reduction of half or more for the lowest concentration. Nevertheless, the calculated concentrations were linearly related to the expected loaded concentrations.

PnTX-G and PnTX-H largely crossed the in vitro intestinal barrier after 2 h, with an amount of toxin crossing the Caco-2 cell monolayers ranging from 15 to 23% and from 22 to 31%, respectively (Table 1). The amount even increased after 6 h, reaching between 34 and 43% for PnTX-G and between 48 and 58% for PnTX-F. The percentage of crossing did not appear to be concentration-dependent. Consistently, a decrease in these two toxins’ amount in the apical compartment was observed, but only at 6 h (80–70% of the loaded amount). Again, this discrepancy could be related to the quantification issue for T0 samples. Indeed, as noticed for PnTX-F, the concentrations measured were far lower than the putative loaded concentrations: around 20% for PnTX-G and even below this (12.5%) for PnTX-H. A linear relationship between the quantified concentrations and the putative ones was observed for both PnTX-G and PnTX–H.

Portimine showed a different behavior as a concentration effect was noticed. Therefore, the crossing of portimine at 2 h increased from 9% of the toxin amount for the lowest concentration tested to 23% for the highest concentration. The values of the crossing were also higher after 6 h, reaching 24 to 39% with increasing concentrations of toxin. A large decrease in the portimine amount in the apical compartment was observed at 2 h (60–40% of the loaded amount) and 6 h (40–29% of the loaded amount). Unlike the PnTXs tested, the crossing of portimine was both concentration- and time-dependent.

The apparent permeability (Papp) values showed that there is no impact of the concentration for all the PnTXs tested, while a concentration-dependent effect was obtained with portimine (Table 2). However, different behaviors could be displayed over time, and two groups could be distinguished. While the values were rather similar at 2 and 6 h for PnTX-A and PnTX-E (means ranging from 1.25 to 2.13 and from 1.49 to 3.75, respectively), higher values at 6 h than at 2 h were observed for the three others, namely PnTX-F, PnTX-G and PnTX-H.

### 2.4. In Silico Predictions 

Various parameters related to the intestinal barrier crossing have been generated using in silico modeling. The first parameters related to bioavailability are the log of the partition coefficient between octanol and water (logP) and Lipinski index alerts (Table 3). LogP values showed that portimine is the most hydrophilic with predicted values ranging from 0.62 to 2.89 (mean of 1.878). In contrast, the more lipophilic PnTXs have values ranging from 3.1 to 7.58. If we take into account only the means, the lipophilicity increases according to the alphabetic letter, from 4.25 for PnTX-A to 5.83 for PnTX-H. Both PnTXs and portimine largely adhered to Lipinski’s Rule of Five, with only a few parameters falling outside the range. Specifically, the prediction indicated that only PnTX-E and PnTX-H had two properties that did not meet the criteria, while portimine had no violations of the rules.

When considering parameters of intestinal absorption, whether predicted by Caco-2 or the Madin–Darby Canine Kidney (MDCK) cell models, it appears that the Absorption, Distribution, Metabolism, Excretion, and Toxicity (ADMET) lab 3.0 prediction seems to fit better with our observations, as both PnTX-A and -E have a lower value than the other PnTXs (Table 4). Portimine was predicted to be the best absorbed, although the difference with the PnTX-F, PnTX-G, and PnTX-H was rather low compared to our results. The AdmetSAR3.0 also separates PnTX-A and PnTX-E from the other PnTXs. However, the absorption of portimine was predicted to be low using this tool, which is not consistent with our results.

The predictions generated with the small-molecule pharmacokinetics prediction (PKCSM) tool were globally not consistent with the two other tools estimating the % of absorption. The predictions of the PKCSM, concluding a high absorption, were only consistent with the in vitro data for PnTX-H and portimine.

## 3. Discussion

All of the compounds tested showed some impairment of the intestinal barrier, at least for the highest concentration tested (32 ng/mL) and at the longer time of exposure (24 h). Both markers (TEER and LY) that were carried out during this in vitro study to follow the local impact on the intestine gave the same kind of results.

The alteration of the Caco-2 monolayer observed with PnTX-G at 24 h for 16 and 32 ng/mL indicates that cells can be lost due to detachment from the support, suggesting that the intestinal epithelium could be impaired. In fact, mice orally treated with 300 μg/kg PnTX-G and above showed alteration of the small intestine. At these doses, all the animals showed villous atrophy with a moderate focal degeneration of the epithelial cells at the tips of the villi [50]. Although the authors claimed that the toxin could have affected the nicotinic receptors present in the intestine and consequently induced some alteration in the gut, such an explanation is not valuable in our case. Unfortunately, we could not provide any clue on how the PnTXs could impair the Caco-2 permeability. We could expect from our results that PnTX-E and PnTX-F could also alter the intestinal epithelium. However, no macroscopic lesions on the intestine, as well as any other organ and tissue, were noticed in mice with the two toxins after oral exposure [23], although this does not exclude the presence of microscopic alterations. Unfortunately, except for [50], the other few in vivo oral studies with PnTXs did not investigate the effect on the intestine after PnTX oral administration [23,25]. It is worth mentioning that the most toxic analogs according to Takada et al. (2001) [51], PnTX-B and PnTX-C, were not tested in our study. For portimine, the impairment of the intestinal monolayer could be due to cell death, as this toxin has been reported as a potent apoptosis inducer [33].

Our results on the crossing of PnTX-G using the Caco-2 monolayer are consistent with the data published from in vivo studies. Indeed, since respiratory failure is the sign of acute oral toxicity in rodents [23,50], it suggests that PnTXs are able to cross the intestinal barrier. Moreover, recently, a kinetic study showed that six hours after the gavage of rats with [H^3^] 100 µg/kg PnTX-G, radioactivity was distributed in the peripheral organs, such as the liver, kidney, and spleen, and also in various brain regions, demonstrating the crossing of PnTX-G [25]. In the same study, the crossing of the placental and blood–brain barriers by PnTX-G has been also reported.

Such impairment could also affect the uptake of other compounds present in food, thus increasing their systemic level and effect. Moreover, the recurrent alteration of the intestinal epithelial integrity could generate long-term effects. Considering the high level that the toxins, especially PnTX-G, can reach in shellfish, the scenario of the concentrations tested is not overestimated.

If, for PnTX-A, PnTX-E and portimine, the quantification values at T0 were close to the loaded concentrations, this was not the case for PnTX-F, PnTX-G, and PnTX-H, as the T0 values measured by liquid chromatography-mass spectrometry (LC-MS) were by far much lower than the loaded concentrations (Supplementary Table S2). Nevertheless, the values measured for the increasing concentrations showed a linear trend, thus indicating that the same multiplication factor should be applied to find the loaded values and that it is not concentration-dependent. The inconsistency between the loaded and the measured values for these three toxins could be explained by some binding issues or by the lower stability of these toxins in the buffer at −20 °C for several months. The Papp value of the portimine was dependent on the concentration, indicating that passive diffusion was responsible for the transport of the portimine from the apical to the basolateral compartment. Indeed, passive paracellular diffusion or passive transcellular diffusion are the two mechanisms of passive absorption that do not require any energy. The first one is the most common for small molecules, generally combined with absorption by transport carriers. For passive transcellular diffusion, Fick’s first law applies and depends on the concentration gradient between the lumen (apical side) and the blood (basolateral side) and on the physicochemical properties of the compound [52,53].

Since a drug with a Papp > 1.5 × 10^−6^ cm·s^−1^ is considered to be highly penetrative, our data suggested that PnTX-F, PnTX-G, and PnTX-H have a high oral bioavailability as well as portimine. In contrast, PnTX-A and PnTX-E could be considered compounds with poor permeability. According to the publication of Press and Di Grandi (2008) [54], the Papp values could be assimilated to the human fraction absorbed. In this case, for PnTX-A and PnTX-E with a Papp < (1–2) × 10^−6^ cm·s^−1^, the fraction absorbed (Fa) might range from 0 to 20%. Fa values between 20 to 80% could be expected for PnTX-F for a Papp between 2 and 10 × 10^−6^ cm·s^−1^, while for the other toxins with Papp values > 10 × 10^−6^ cm·s^−1^, a high permeability with 80–100% Fa could be estimated. However, other papers proposed different levels of Papp for estimating permeability, which would rather conclude to a moderate crossing for PnTX-A, PnTX-E, and PnTX-F while all the others would be highly permeable [55]. In silico predictions gave some inconsistent results, between the prediction of the different tools as well as compared to the in vitro results. According to the Lipinski rules, portimine was predicted as highly soluble and highly permeable, which fits rather well with the in vitro data. Except for PnTX-E and PnTX-H which are less compliant, the Lipinski rules predicted that the other analogs are also highly soluble and highly permeable. This prediction does not fit the in vitro data, as PnTX-A and PnTX-E have a low permeability while PnTX-F, PnTX-G, and PnTX-H showed high permeability across the intestinal barrier. In fact, when comparing the Papp values on Caco-2 cells, the prediction for portimine does not fit the results obtained in vitro. In contrast, the predicted Papp was close to the ones calculated for PnTX-A for two of the tools, PKCSM and ADMETlab 3.0. However, the AdmetSAR3.0 provided values for PnTX-A and PnTX-E that were much higher than the ones estimated by in vitro studies. Although the Papp predicted by PKCSM was rather low, the Fa estimated for all the toxins was close to 100%, not reflecting the differences of the predicted Papp values between PnTX-E (0.296) and PnTX-H (2.169) provided by the same tool. Moreover, the predictions with this tool were globally not consistent, with the two other tools estimating the % of absorption. The predictions of PKCSM concluding to a high absorption were only consistent with the predictions of the tool for PnTX-H and portimine. Therefore, it seems that the PKCSM tool was a poor predictor of the fate of the toxins tested in this study.

Overall, based on these in vitro results, there could be expected a higher oral bioavailability for PnTX-F, PnTX-G, and PnTX-H, as well as systemic effects compared to PnTX-A and PnTX-E. This is consistent with the ranking of PnTX analogs based on the oral toxicity data demonstrating a decreasing potency for PnTX-F, PnTX-G/PnTX-H, and PnTX-E [20,21,24]. Moreover, the rapid intestinal absorption observed in the Caco-2 model, within a few hours, is also consistent with the rapid toxicity (within one hour) that has been reported for PnTX-G after oral exposure to a certain dose [50].

However, it is important to acknowledge the limitations of Caco-2 monolayers in providing a reliable estimation of a substance’s oral bioavailability. First, it is well known that the tight junctions of Caco-2 monolayers generate a more restrictive passage compared to the human epithelium [56,57]. Indeed, it has been demonstrated that, due to this property, the permeability derived from Caco-2 cells can under-predict the Fa for compounds with a molecular weight lower than 300 g/mol [58]. As PnTXs and portimine have a higher molecular weight (around 694 and 402 g/mol, respectively), it could be assumed that the results obtained in our study reflect the Fa in humans. Second, the intestinal epithelium is composed of several cell types. As the second most abundant cell type, goblet cells can produce a mucus gel that protects the epithelial cells from contact with food contaminants. Moreover, the addition of goblet cells in cell monolayers (e.g., co-culture of Caco-2 and HT29-MTX cells) reduces the tightness of the tight junctions [59]. Therefore, a protective role of the mucus on the toxicity of substances to the intestinal epithelium and the capacity of intestinal absorption and crossing could not be ruled out. Studies with more complex intestinal cell models, such as co-cultures Caco-2/HT-29-MTX cells or a reconstructed intestinal epithelium [60,61], could be proposed to refine the results obtained with Caco-2 monolayers. Moreover, our results are only qualitative and should be completed with in vivo experimental data to calculate more exactly the oral bioavailability of the different toxins.

Finally, the impact of digestion and microbiota on the toxin can be also worth investigating to describe if any modification can occur after ingestion, as shown for other toxins [62,63,64]. Nevertheless, the rodent study performed with PnTX-G showed that the parent toxin could be detected unchanged in various internal organs, although some metabolites have been detected in the liver [25]. Therefore, it seems that both digestion and the intestinal microbiota are not prone to modify the structure of PnTXs.

## 4. Materials and Methods

### 4.1. Chemicals

A PnTX-G certified reference material, at 1.92 ± 0.09 μg/mL, was purchased from NRCC (Halifax, NS, Canada). PnTX-E, PnTX-F, PnTX-H, and portimine, produced as reference materials by the Cawthron Institute (Nelson, New Zealand), were provided by Novakits (Nantes, France). For the cell culture assays, synthetized PnTX-A was given by Dr. A. Zakarian, while a PnTX-A reference material, concentrated to 5.0 μg/mL, was obtained from Abraxis LLC (Warminster, PA, USA) for liquid chromatography–mass spectrometry (LC-MS) analyses.

Cell culture products, Hank’s Balanced Salt Solution (HBSS), and 4-(2-hydroxyethyl)-1-piperazineethanesulfonic acid (HEPES) were purchased from Gibco (Cergy-Pontoise, France). Bovine serum albumin (BSA) and Lucifer Yellow CH di-lithium (LY) were supplied by Sigma-Aldrich (Saint Quentin Fallavier, France).

For the LC-MS analyses, all solutions were prepared using chemicals of LC-MS grade and ultrapure water (18.2 MΩ·cm) obtained by purifying distilled water through a Milli-Q system. Acetonitrile, methanol, and formic acid were procured from Fisher Scientific (Loughborough, UK). Ultra-pure-grade carrier nitrogen (N2, 99.999% pure) was purchased from Linde Gas (Montereau-Fault-Yonne, France).

### 4.2. Cells

#### 4.2.1. Cell Culture

All experiments were performed with human intestinal Caco-2 cells (ATCC-HTB37), acquired from the American Type Culture Collection (ATCC; Manassas, VA, USA). Caco-2 cells originate from a colorectal adenocarcinoma, but upon reaching confluence, they differentiate and express the characteristics of enterocytes. The cells were cultivated in minimum essential medium containing 5.5 mM D-glucose, Earle’s salts, and 2 mM L-alanyl-glutamine (MEM GlutaMAX™, Gibco, Bromont, QC, Canada) supplemented with 10% fetal bovine serum (FBS, Gibco), 1% non-essential amino acids, 50 U/mL penicillin, and 50 μg/mL streptomycin (Gibco) under humidified conditions (37 °C, 5% CO_2_, 95% humidity). The cells were grown for 3–4 days up to 80% confluence before trypsinization and sub-culture. For the experiments, the cells were seeded in TRANSWELL^®^ plates (12 wells, 0.4 µm pore size; Costar^®^, Corning Incorporated, Corning, NY, USA) at a density of 90,000 cells/cm^2^. Prior to toxin treatment, highly differentiated Caco-2 cells were obtained after 24–26 days of culture, with medium renewal every 2–3 days. Passage numbers between 30 and 40 were used for experiments.

#### 4.2.2. Cell Treatment

PnTX-A, PnTX-G, PnTX-E, PnTX-H, and portimine stock solutions were prepared in methanol, while PnTX-F was provided in acetonitrile/acetic acid (50%/0.1%). For each toxin, the cells were incubated at 8, 16, and 32 ng/mL (diluted in HBSS) for 2 h, 6 h, and 24 h, with a final volume of 1.5% solvent. The controls were incubated with the cell culture medium containing the respective solvent of the toxin at 1.5%. Each experiment included three biological replicates. Two to three independent experiments were conducted.

#### 4.2.3. Barrier Integrity

- Transepithelial electrical resistance (TEER)

Epithelial integrity was examined by measuring the TEER between the apical and basolateral compartments. After rinsing with a HBSS solution (HBSS, HEPES 5 mM, pH 7.4), the Caco-2 monolayers were equilibrated for 30 min at 37 °C with 300 and 1000 µL HBSS in the apical (A) and basolateral (B) chambers, respectively. Then, the content of the apical compartment was replaced with 500 µL HBSS-containing toxins. TEER was quantified with a Millipore Millicell Electrical Resistance System (ERS Millipore, EMD Millipore Corporation, Burlington, MA, USA). The mean value of two measurements per well before and after the treatment was calculated and multiplied by the surface of the insert to obtain Ω × cm^2^. After blank subtraction, the results were presented as a percentage of the solvent control.

- Lucifer Yellow

Lucifer Yellow (LY) was also used to ensure the integrity of the monolayers, as this marker shows paracellular permeability when cellular tight junctions are affected. For the assay, both compartments of the insert system were washed with Hank’s balanced salt solution (HBSS). Then, 0.5 mL of 0.1 mg/mL Lucifer Yellow in HBSS buffer and 1 mL of pure HBSS buffer were added to the apical and basolateral compartments, respectively. After a 2 h incubation at 37 °C, the media of the basolateral compartments were collected and the fluorescence of each compartment was measured in triplicate (excitation: 405 nm, emission: 520 nm) with a FLUOstar Optima microplate reader (BMG Labtech, Ortenberg, Germany). After subtraction of the blank (HBSS), the permeability of the toxin was calculated against the value obtained for pure LY and reported as % permeability.

#### 4.2.4. Barrier Permeability of Toxins

The apical and basolateral compartments were collected after 2, 6, and 24 h treatment time. Parts of the solutions that were used to load the apical compartments were also stored (T0) to take into account the real concentration and not the estimated concentration. All the samples were stored at −20 °C until LC/MS analysis.

- Liquid Chromatography coupled to tandem Mass Spectrometry (LC-MS/MS) analysis

The LC-MS/MS analysis of toxins was implemented for the quantitative analysis of toxins. The LC system was an Agilent 1200 (Agilent Technologies, Santa Clara, CA, USA). The chromatographic separation was performed using a column Hypersil GOLD^®^ C18 50 mm × 2.1 mm, 1.9 μm (Thermo Scientific, Waltham, MA, USA). Eluent A consisted of water, and eluent B of acetonitrile/water at a ratio of 95:5 (*v*/*v*), with both eluents containing 50 mM formic acid and 2 mM ammonium formate. The LC flow rate was 0.4 mL/min, and the gradient was programmed as follows: 10% B held for 0.5 min, 10–90% B in 5 min, 90% B held for 1 min, 90–10% B in 0.5 min, and 10% B held for 3.0 min, for a total run time of 10 min. The injection volume was 5 μL, while the column temperature was maintained at 40 °C. The column effluent was transferred via a divert valve (Valco Instruments Co., Inc., Houston, TX, USA), either to the mass spectrometer (between 1.7 and 5.5 min) or to waste.

The detection system was constructed with an API 4000 triple quadrupole mass spectrometer (Sciex, Framingham, MA, USA) equipped with an ElectroSpray Ionization (ESI) source (Turbo V). The mass spectrometer was operated in positive ESI mode. The source temperature was set at 600 °C, and the spray voltage was 5500 V. Air was used as a nebulizer gas and an auxiliary gas, with a gas pressure of 40 and 60 psi, respectively. Meanwhile, the curtain gas and collision gas were nitrogen, with a gas pressure of 15 and 8 psi, respectively. The mass spectrometer was operated in multiple reaction monitoring mode (MRM). One transition was used for quantification (Q) and another as a qualifier transition (q). The optimized compound-dependent parameters are listed in Table S1. A mass resolution of 0.7 Da full width at half maximum (FWHD) was set on the first (Q1) and the third (Q3) quadrupoles. Instrument control and data were handled with Analyst software version 1.5.1 (Sciex, Framingham, MA, USA).

The portimine and PnTX retention times varied between 2.7 and 4.4 min (Figure S1). To ensure reliable results, the samples were analyzed using bracketing calibration curves composed of 9 levels ranging between 0.05 and 45 ng/mL and prepared in HBSS. The determination coefficient (r^2^) of the calibration curves had to be ≥0.98. LOQ was defined as the lowest level of the calibration curve with a signal-to-noise ratio higher than 10 for the quantitative and qualifier transition, which is 0.05 ng/mL for all toxins.

- Calculation of the apparent permeability Papp

The apparent permeability coefficient (Papp) was calculated using the following equation:

Papp = (dQ/dt)/(S × C0) (1)

where dQ/dt represents the amount of toxin transported within a given time period, C0 represents the initial concentration of toxin measured into the donor compartment, and S is the surface area of the cell monolayer.

#### 4.2.5. In Silico Tools

Several parameters relevant to intestinal permeability have been predicted using quantitative structure analysis relationship (QSAR) algorithms. LogP, the partition coefficient between water and n-octanol, is a measure of molecule solubility and a marker of bioavailability. The higher the value is, the more lipophilic the substance is, with higher capacities for bioaccumulation up to a certain point. Absorption across the intestinal barrier can be also evaluated using algorithms based on databases compiling the results from assays performed using parallel artificial membrane permeability (PAMPA), intestinal Caco2 cells, or kidney MDCK cells [65]. The human intestinal effective permeability (Peff) is a parameter [66]. The Lipinski index is based on the physico-chemical characteristics of molecules. It is known as ‘the rule of 5′, predicting that poor absorption or permeation is more likely when there are more than 5 H-bond donors, 10 H-bond acceptors, the molecular weight (MWT) is greater than 500, and the calculated Log P (CLogP) is greater than 5 (or MlogP > 4.15) [49].

The predictions of the parameters were performed for the different toxins using several free types of QSAR software that differ regarding database or rules and associated algorithms. ADMETlab 3.0 (https://admetlab3.scbdd.com/, accessed on 6 December 2024) is an upgrade of the previous version ADMET lab 2.0 [67], expanding the dataset to 375,187 data points covering 88 endpoints, including 30 new endpoints and updates to 4 existing ones. The new endpoints cover absorption, distribution, metabolism, toxicity, physicochemical properties, and drug chemistry. AdmetSAR3.0 (https://lmmd.ecust.edu.cn/admetsar3/index.php, accessed on 6 December 2024) is a significantly enhanced version of the widely used comprehensive ADMET assessment tool, admetSAR2.0 [68]. PKCSM (https://biosig.lab.uq.edu.au/pkcsm/, accessed on 6 December 2024) is a valuable tool to help medicinal chemists to find the balance between potency, safety, and pharmacokinetic properties [69]. Compared to the free web-based tools for ADME and pharmacokinetics (e.g. PKCSM and AdmetSAR), the SwissADME web tool (http://www.swissadme.ch, accessed on 6 December 2024) uses different input methods and can compute multiple molecules [70].

#### 4.2.6. Statistics

GraphPad Prism software (version 9.5.1) was used for statistical analyses. An analysis of the variance (ANOVA) was performed, and when the effect of concentration was significant (*p*-value < 0.05), the values were compared to the control using Dunnett’s test.

## 5. Conclusions

Our results highlighted a difference in behavior depending on the toxin of the pinnatoxin group, as already described in in vivo studies. Two PnTX analogs (PnTX-G and -H) showed a slight effect on the intestinal barrier integrity compared to the four other toxins (PnTX-A, -E, -F, and portimine) that could alter the intestinal epithelium. Moreover, while PnTX-A and PnTX-E displayed poor permeability, the other ones could easily cross the intestinal barrier. Finally, a passive diffusion mechanism could even be inferred for portimine. Our results suggest that some analog(s) should be more prone to be distributed in the body and, consequently, should be prioritized for further investigation on public health.

## Figures and Tables

**Table 1:** Percentage of toxin crossing the Caco-2 cell monolayers following 2 and 6 h treatment. Transport experiments were performed by loading the apical compartments (A to B) with three concentrations of toxin. Percentage values are presented as mean ± SD. The crossing was calculated considering the quantified amount of the loaded concentrations (T0 samples). Three independent experiments were performed (excluding PnTX-E and PnTX-F, with two independent experiments). * result available for only one replicate.

Toxin	Time (h)	Loaded Concentration (ng/mL)		
		8	16	32
PnTX-A	2	2.35 ± 1.66	2.26 ± 0.34	2.01 ± 0.63
	6	3.44 ± 0.62	3.10 ± 1.53	2.39 ± 0.65
PnTX-E	2	6.05 *	2.41 ± 0.65	2.92 ± 0.45
	6	3.29 ± 0.64	2.40 ± 1.06	3.85 ± 1.15
PnTX-F	2	14.43 ± 2.73	10.12 ± 3.82	11.20 ± 3.27
	6	26.00 ± 0.52	18.36 ± 4.64	22.09 ± 1.76
PnTX-G	2	15.62 ± 5.24	23.32 ± 4.36	23.07 ± 5.56
	6	34.43 ± 9.39	36.83 ± 5.26	43.41 ± 6.15
PnTX-H	2	31.52 ± 5.07	22.05 ± 7.80	29.07 ± 3.54
	6	58.06 ± 8.71	48.83 ± 11.22	54.22 ± 3.91
Portimine	2	9.23 ± 1.46	15.48 ± 0.89	23.25 ± 6.88
	6	24.23 ± 3.69	30.63 ± 5.62	39.45 ± 4.86

**Table 2:** Apparent permeability (Papp) of toxins, following 2 and 6 h treatment of Caco-2 cell monolayers. Transport experiments were performed by loading the apical compartments (A to B) with three concentrations of toxin. Papp values are presented as mean ± SD and expressed in 10^−6^ cm·s^−1^. Three independent experiments were performed (excluding PnTX-E and PnTX-F, with two independent experiments). * result available for only one replicate.

Toxin	Time (h)	Loaded Concentration (ng/mL)		
		8	16	32
PnTX-A	2	1.45 ± 1.03	1.40 ± 0.21	1.25 ± 0.39
	6	2.13 ± 0.38	1.92 ± 0.95	1.48 ± 0.41
PnTX-E	2	3.75 *	1.50 ± 0.40	1.81 ± 0.28
	6	2.04 ± 0.40	1.49 ± 0.65	2.38 ± 0.71
PnTX-F	2	8.95 ± 1.69	6.28 ± 2.37	6.94 ± 2.03
	6	16.12 ± 0.24	11.38 ± 2.88	13.70 ± 1.09
PnTX-G	2	9.69 ± 3.25	14.45 ± 2.70	14.31 ± 3.45
	6	21.35 ± 5.82	22.84 ± 3.26	26.92 ± 3.82
PnTX-H	2	19.54 ± 3.14	13.67 ± 4.83	18.03 ± 2.19
	6	35.99 ± 5.40	30.28 ± 6.69	33.62 ± 2.43
Portimine	2	5.72 ± 0.91	9.60 ± 0.55	14.42 ± 5.27
	6	15.02 ± 2.29	18.99 ± 349	24.46 ± 3.01

**Table 3:** LogP and Lipinski values predicted by quantitative structure–activity relationships (QSARs).

	Toxin	PnTX-A	PnTX-E	PnTX-F	PnTX-G	PnTX-H	Portimine
Canonical SMILE		CC1CC23CCC(=CC2C4C5C(C(CC(O4)(O5)CC6CCCC7(O6)CCC8(O7)C(CCC(O8)CC(=C)CCCC3=NCC1C)(C)O)C)O)C(=O)O	CC1CC2C3C4C=C(CCC45CC(C(CN=C5CCCC(=C)CC6CCC(C7(O6)CCC8(O7)CC(C(C(O8)CC(C1)(O2)O3)O)C)(C)O)C)C)C(CCC(=O)O)O	CC1CC2C3C4C=C(CCC45CC(C(CN=C5CCCC(=C)CC6CCC(C7(O6)CCC8(O7)CC(C(C(O8)CC(C1)(O2)O3)O)C)(C)O)C)C)C9CCC(=O)O9	CC1CC23CCC(=CC2C4C5C(C(CC(O4)(O5)CC6CCCC7(O6)CCC8(O7)C(CCC(O8)CC(=C)CCCC3=NCC1C)(C)O)C)O)C=C	CC1CC2C3C4C=C(CCC45CC(C(CN=C5CCCC(=C)CC6CCC(C7(O6)CCC8(O7)CC(C(C(O8)CC(C1)(O2)O3)O)C)(C)O)C)C)C=C	CC1CC(C(=O)C2C3C=C(CCC34CCN=C4C(CC5(O2)CCC1O5)O)C=C)O
Solubility (logP)	ADMETlab 3.0	3.62	3.47	3.78	4.88	5.16	0.62
	AdmetSAR3.0	3.22	4.20	4.91	5.47	6.12	2.19
	SwissADME/iLogP	4.41	4.80	5.25	5.63	5.52	2.73
	SwissADME/XLogP3	4.36	4.10	4.89	5.44	5.88	1.44
	SwissADME/WLOGP	6.23	6.62	7.10	7.33	7.58	1.95
	SwissADME/MLogP	3.20	3.10	3.86	4.10	4.27	1.32
	SwissADME/Silicos-IT	4.69	5.29	5.78	6.15	6.28	2.89
Lipinski	SwissADME	1	2	1	1	2	0

SMILE: Simplified molecular input line entry. IlogP: in-house physics-based method implemented from [45]. XLogp3: atomistic and knowledge-based method calculated by XlogP program, version 3.2.2, courtesy of CCBG, Shanghai Institute of Organic Chemistry. WLogP: atomistic method implemented from [46]. MLogP: topological model implemented from [47,48,49]. Silicos-IT: hybrid fragmental/topological method calculated by Filter-IT program, version 1.0.2, courtesy of Silicos-IT, https://www.silicos-it.be (accessed on 6 December 2024).

**Table 4:** Parameters and levels of intestinal permeability predicted by various QSARs.

	Toxin	PnTX-A	PnTX-E	PnTX-F	PnTX-G	PnTX-H	Portimine
Caco2 Papp (10^−6^ cm·s^−1^)	PKCSM	1.89	0.30	2.08	2.1	2.17	1.09
	ADMETlab 3.0	5.2	5.18	9.39	8.83	8.77	9.54
	AdmetSAR3.0	1.04	0.72	2.31	3.71	3.73	1.24
MDCK	ADMETlab 3.0 (10^−6^ cm·s^−1^)	11.34	10.58	17.56	15.46	16.49	18.74
	AdmetSAR3.0	low	low	low	low	low	high
PAMPA (logPeff)	ADMETlab 3.0	0.954	0.256	0.017	0.023	0.001	0.415
Intestinal absorption	AdmetSAR3.0	low	low	low	low	high	high
	ADMETlab3.0	low	low	low	low	low	low

## Data Availability

Data will be made available on request.

## Supplementary Materials

The following supporting information can be downloaded at https://www.mdpi.com/article/10.3390/md23010026/s1: Figure S1: LC-MS/MS toxin profile obtained from the calibration curve level at 4 ng/mL. For each toxin, the peak with the highest intensity corresponds to the quantitative transition; Table S1: compound-dependent tandem mass spectrometry parameters; Table S2: T0 concentrations quantified by LC/MS-MS; Table S3: % theoretical amount of toxin crossing the Caco-2 cell monolayers following 2 and 6 h treatment.
