# Supplementary material for: A Comparative Study of the In Vitro Intestinal Permeability of Pinnatoxins and Portimine
Source: Mar Drugs. 2025 Jan 7;23(1):26. doi: 10.3390/md23010026 (PMC11766601; doi:10.3390/md23010026)
Supplement: Supplementary file 1 [file marinedrugs-23-00026-s001.zip › marinedrugs-3315637-supplementary.pdf]

Supplementary figure

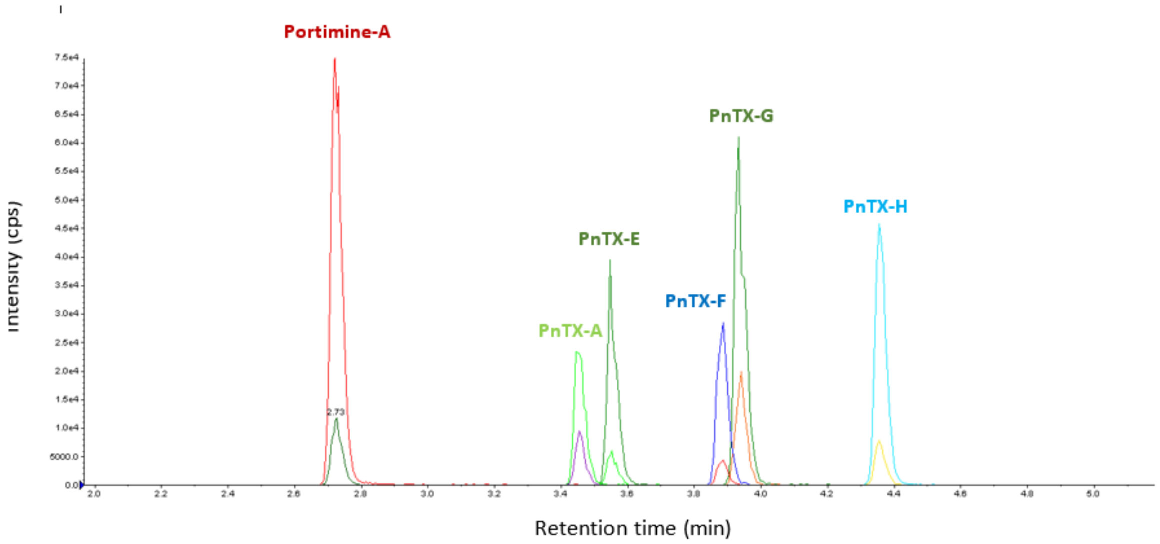

Figure S1: LC-MS/MS toxin profile obtained from the calibration curve level at 4 ng/mL. For each toxin, the peak with the highest intensity corresponds to the quantitative transition.

Supplementary tables

Table S1: Compound-dependent tandem mass spectrometry parameters.

Compound	Precursor ion (m/z)	Product ion * (m/z)	Collision energy (V)
PnTX-A	712.5	164.2 (Q)	77
		458.3 (q)	61
PnTX-E	784.4	164.2 (Q)	77
		488.3 (q)	61
PnTX-F	766.4	164.2 (Q)	77
		488.3 (q)	61
PnTX-G	694.5	164.2 (Q)	77
		458.3 (q)	61
PnTX-H	708.5	164.2 (Q)	77
		488.3 (q)	61
Portimine	402.2	384.2 (Q)	22
		246.2 (q)	28

* Q: Quantitative transition; q: qualifier transition

Table S2: T0 concentrations quantified by LC/MS-MS

	Loaded concentration (ng/mL)		
**Toxin**	**8**	**16**	**32**
PnTX-A	9.58 ± 0.81	17.73 ± 0.35	41.47 ± 2.57
PnTX-E	7.00 ± 0.08	13.95 ± 0.92	27.15 ± 0.78
PnTX-F	2.70 ± 0.71	6.81 ± 2.57	15.15 ± 5.30
PnTX-G	3.76 ± 1.30	5.78 ± 0.30	12.32 ± 2.12
PnTX-H	2.03 ± 0.23	4.74 ± 0.65	9.43 ± 1.58
Portimine	7.38 ± 0.10	13.83 ± 2.17	28.13 ± 2.27

Table S3: % theoretical amount of toxin crossing the Caco-2 cell monolayers following 2 and 6 h treatment. Transport experiments were performed by loading the apical compartments (A to B). % values are presented as mean ± SD. The crossing was calculated considering the theoretical loaded concentrations (8, 16 and 32 ng/mL). Three independent experiments were performed (excluding PnTX-E and PnTX-F with two independent experiments). result available for only one replicate*

	Time (h)	Loaded concentration (ng/mL)		
**Toxin**		**8**	**16**	**32**
PnTX-A	2	2.83 ± 2.1	2.5 ± 0.33	2.60 ± 0.80
	6	4.08 ± 0.52	3.42 ± 1.63	3.12 ± 1.05
PnTX-E	2	5.25*	2.12 ± 0.71	2.47 ± 0.31
	6	2.87 ± 0.53	2.12 ± 1.06	3.25 ± 0.88
PnTX-F	2	4.75 ± 0.35	4.00 ± 0.00	5.03 ± 0.31
	6	8.75 ± 2.12	7.44 ± 0.97	10.31 ± 2.83
PnTX-G	2	6.83 ± 1.04	8.37 ± 1.19	8.64 ± 0.53
	6	15.17 ± 0.58	13.25 ± 1.30	16.58 ± 2.86
PnTX-H	2	3.96 ± 0.19	3.21±0.94	4.32 ± 1.10
	6	7.37 ± 1.42	7.10 ± 0.79	8.04 ± 1.80
Portimine	2	8.50 ± 1.25	13.33 ± 1.69	20.73 ± 7.28
	6	22.33 ± 3.36	26.08 ± 3.00	34.60 ± 4.41
